# Nasal High-Frequency Oscillatory Ventilation vs. Nasal Continuous Positive Airway Pressure as Therapy for Postextubation Respiratory Failure in Infants After Congenital Heart Surgery

**DOI:** 10.3389/fped.2021.700632

**Published:** 2021-08-16

**Authors:** Hong-Lin Wu, Yu-Qing Lei, Wen-Peng Xie, Qiang Chen, Yi-Rong Zheng

**Affiliations:** ^1^Department of Cardiac Surgery, Fujian Branch of Shanghai Children's Medical Center, Fuzhou, China; ^2^Fujian Children's Hospital, Fuzhou, China; ^3^Fujian Maternity and Child Health Hospital, Affiliated Hospital of Fujian Medical University, Fuzhou, China; ^4^Fujian Key Laboratory of Women and Children's Critical Diseases Research, Fujian Maternity and Child Health Hospital, Fuzhou, China

**Keywords:** nasal high-frequency oscillatory ventilation, non-invasive ventilation, post-extubation respiratory failure, infants, congenital heart surgery

## Abstract

**Objective:** This study aimed to evaluate the effects of nasal high-frequency oscillatory ventilation (NHFOV) vs. nasal continuous positive airway pressure (NCPAP) on postextubation respiratory failure (PRF) in infants after congenital heart surgery (CHS).

**Method:** Eighty infants underwent postoperative invasive mechanical ventilation for more than 12 h and planned extubation. The infants were randomized to undergo either NHFOV or NCPAP after extubation. Primary outcomes were the incidence of PRF and reintubation, the average PaCO_2_ level, the average oxygenation index (OI), and pulmonary recruitment in the early extubation phase. Secondary outcomes included the NCPAP/NHFOV time, length of hospital stay, treatment intolerance, signs of discomfort, pneumothorax, adverse hemodynamic effects, nasal trauma, and mortality.

**Results:** Except for PaCO_2_ within 12 after extubation (39.3 ± 5.8 vs. 43.6 ± 7.3 mmHg, *p* = 0.05), there was no statistically significant difference for any of the primary outcome measure (PRF, reintubation within 12 h after extubation, oxygenation index within 12 h after extubation, or lung volumes on X-ray after extubation) or secondary outcome measures (duration of non-invasive ventilation, duration of hospital stay, ventilation intolerance, signs of discomfort, pneumothorax, nasal trauma, adverse hemodynamic effects, or death prior to discharge), *p* > 0.1 for each comparison.

**Conclusion:** NHFOV therapy after extubation in infants after CHS was more efficient in improving CO_2_ cleaning than NCPAP therapy, but there was no difference in other outcomes (PRF, reintubation, oxygenation index, and pulmonary recruitment).

## Introduction

In infants who undergo congenital heart surgery (CHS), cardiopulmonary bypass, acute cardiogenic pulmonary edema, and pulmonary infection may contribute to an increased risk of postextubation respiratory failure (PRF) (1). Non-invasive ventilation (NIV) provides benefits to infants postextubation and post-infectious pulmonary disease, reducing the risk for PRF (2). Nasal continuous positive airway pressure (NCPAP) is a widely used respiratory support after CHS, but 40% of infants and neonates with NCPAP do not achieve improved oxygenation and are difficult to wean from invasive mechanical ventilation (IMV). In addition, NCPAP is associated with the occurrence of respiratory acidosis related to inadequate expiratory flow (3, 4). A new mode of NIV, nasal high-frequency oscillatory ventilation (NHFOV), has emerged in recent years as a technique to combine the advantages of both invasive HFOV and NCPAP (5). Theoretically, NHFOV should reduce the risk of hypercapnic respiratory failure compared to NCPAP, but its application in infants needs further research. To date, few studies have focused on the use of NHFOV in infants with CHS who underwent surgical correction. Therefore, we hypothesized that NHFOV would be advantageous in treating infant patients with PRF. This study aimed to evaluate the effects of NHFOV vs. NCPAP on PRF in infants after CHS.

## Materials and Methods

### Sample Size

The sample size was determined with G Power 3.1.9.2. The alpha value was set as 0.05 and the power as 0.80. Then, according to the effect size, we calculated that the minimum sample size of the case group should be 40. Considering a 20% drop rate, we selected 50 patients as the case group. According to the 1:1 ratio between the case group and the control group, 50 other patients were selected as the control group.

### Population and Study Design

We performed this prospective, randomized study in infants who underwent CHS at the pediatric cardiac intensive care unit from January 2020 to January 2021. The study was approved by the ethics committee of our hospital (NO. 2020YJ181) and adhered to the tenets of the Declaration of Helsinki (as revised in 2013). Written informed parental consent was obtained for all subjects. Clinical data for all patients are shown in Table 1.

**Table 1:** Clinical data of all patients.

**Items**	**NCPAP**	**NHFOV**	***p*-value**
	**(*n* = 40)**	**(*n* = 40)**	
Age at extubation, months	2.3 ± 0.4	2.2 ± 0.4	0.17
Weight at extubation, kg	4.4 ± 0.5	4.2 ± 0.5	0.18
Male, *n*	21	18	0.52
Infants with CPB, *n*	30	28	0.61
Duration of CPB, h	0.8 ± 0.1	0.8 ± 0.2	0.58
Duration of cardiac surgery, h	2.3 ± 0.5	2.3 ± 0.4	0.77
Duration of IMV support, h	32.2 ± 6.0	34.0 ± 7.2	0.22
The final PaCO_2_ levels before extubation, mmHg	42.3 ± 5.0	40.6 ± 5.1	0.12
The final OI before extubation, mmHg	230 ± 32	237 ± 42	0.39
Lung volumes before extubation, median (IQR), rids	8 (7–8)	8 (7.25–8)	0.84
**Diagnosis**			
PDA, *n*	10	9	1.00
VSD/ASD, *n*	23	24	1.00
PS, *n*	2	3	1.00
TAPVC, *n*	4	2	0.67
CoA, *n*	1	2	1.00

*NHFOV, nasal high-frequency oscillatory ventilation; NCPAP, nasal continuous positive airway pressure; CPB, cardiopulmonary bypass; IMV, invasive mechanical ventilation; OI, oxygenation index; PDA, patent ductus arteriosus; VSD, ventricular septal defect; ASD, atrial septal defect; PS, pulmonary artery stenosis; TAPVC, total anomalous pulmonary venous drainage; CoA, coarctation of aorta*.

The inclusion criteria were as follows: (1) infants were receiving postoperative invasive mechanical ventilation for more than 12 h and were ready for planned extubation, and (2) their anatomical correction was satisfactory, cardiac function had recovered well after CHS, and hemodynamics were stable. Exclusion criteria were as follows: (1) patients with difficulty weaning from IMV, (2) contraindication to NIV, (3) accidental or self-extubation, and (4) parents' decision not to participate.

### NIV Interventions

NCPAP was delivered through a time-cycled, pressure-limited, and continuous-flow ventilator (Infant Flow SiPAP system, CareFusion, California, USA). The settings in the NCPAP group were as follows: PEEP at 3–8 cm H_2_O; oxygen flow at 5–10 L/min; and FiO_2_ adjusted to obtain adequate oxygenation (SpO_2_ > 90%).

NHFOV support was delivered through a neonatal ventilator (SLE 5000, SLE UK, Croyden, United Kingdom) generated by piston/membrane oscillators. The settings in NHFOV treatment were as follows: frequency at 10 Hz; inspiratory:expiratory ratio of 1:1; PEEP at 5–10 cm H_2_O; amplitude at 25–40 cm H_2_O; and FiO_2_ adjusted to obtain adequate oxygenation (SpO_2_ > 90%).

The NIV treatment was considered to stop according to the following criteria: NCPAP: pressure support at <4 mm H_2_O; FiO_2_ at <40%. NHFOV: pressure support at <4 mm H_2_O; amplitude at <20 mm H_2_O; FiO_2_ at <40%.

After extubation, all infants were supported with NCPAP or NHFOV through silicone binasal prongs. If they met the criteria for reintubation, NCPAP or NHFOV treatment was stopped, and reintubation was encouraged. If they met the criteria for stopping NIV treatment, they would transfer from NCPAP or NHFOV therapy to conventional oxygen therapy. Both groups received similar treatments in medicine, nursing, and respiratory management.

### Diagnostic Criteria

The diagnostic criteria for PRF were the presence and persistence of any of the following: respiratory acidosis (pH <7.25 with PaCO_2_ > 50 mm Hg), SpO_2_ < 90% or PaO_2_ < 60 mm Hg, tachypnea, increased work of breathing, such as retractions, grunting, head bobbing, nasal flaring, or belly breathing (6). Pulmonary recruitment was defined as sustained airway pressure with high levels of positive end-expiratory pressure to favor homogeneous pulmonary ventilation and oxygenation (7). The criteria for reintubation were as follows: severe hypoxemia (PaO_2_ < 5 0 mmHg), severe hypercapnia (PaCO_2_ > 75 mm Hg), dyspnea (>60 min^−1^), or elevated serum lactic acid (>2.0 mmol/L) (8).

### Data Collection

The researchers randomly divided eligible patients into the NCPAP group and the NHFOV group (leaflet group) based on computer-generated random numbers. Baseline characteristics were recorded, including age, weight, sex, infants with cardiopulmonary bypass (CPB), CPB time, surgical time, IMV time, final PaCO_2_ levels, final oxygenation index (OI) levels, and lung volumes before extubation. From pre-extubation to 12 h after extubation, blood gas analysis was evaluated at 4-h intervals. Chest radiography was used to evaluate pulmonary inflation before extubation and 12 h after extubation. An electrocardiogram monitor was used to record the oxygenation information after extubation.

### Outcomes and Termination

The primary outcomes were as follows: the incidence of PRF and reintubation, the effects on the PaCO_2_ level and the OI, and pulmonary recruitment in the early extubation phase (within 12 h after extubation). The secondary outcomes were the difference in the NIV time, the length of hospital stay, and the incidence of pneumothorax, treatment intolerance, signs of discomfort, adverse hemodynamic effects, nasal trauma, and death in the hospital. The endpoints of the study were as follows: (1) stopped NIV treatment according to doctors' suggestions; (2) reintubation; (3) death; and (4) parents' decision not to participate.

### Statistical Analysis

Data analysis was performed using SPSS software, version 25. Independent continuous variables are presented as the mean ± standard deviation (SD). Counts describe the enumeration data. Means were compared using Student's *t*-test, and Fisher's exact test was used for categorical data. The Mann–Whitney *U*-test was applied for non-normally distributed data. A two-sided *p*-value of <0.05 was regarded as statistically significant.

## Results

### Baseline Data

A total of 100 infants were screened in this study, of which eight did not meet the inclusion criteria, three were supported by different NIV modes, and nine parents declined participation. Eighty infants were ultimately enrolled and completed the study: 40 were extubated to NCPAP, and 40 were extubated to NHFOV. There were no significant differences in the main clinical characteristics, including sex, age, weight, surgical time, CPB time, IMV time, final PaCO_2_ levels, final OI levels and lung volumes before extubation, and types of congenital heart diseases. These results indicated that the groups of infants were homogeneous and comparable (Table 1).

### Primary Outcomes

The primary outcomes for NCPAP and NHFOV are shown in Table 2. For infants after extubation, three infants receiving NHFOV treatment vs. nine infants receiving NCPAP treatment experienced PRF (*p* = 0.11). Two infants receiving NHFOV treatment vs. five infants receiving NCPAP treatment needed reintubation in early extubation (*p* = 0.71). NHFOV treatment significantly decreased the PaCO_2_ level within 12 h after extubation (39.3 ± 5.8 mmHg vs. 43.6 ± 7.3 mmHg, *p* = 0.05). NHFOV treatment slightly increased the OI level within 12 h after extubation (226 ± 33 mmHg vs. 210 ± 38 mmHg, *p* = 0.18). The lung volumes was on eight ribs in the NHFOV group and on seven to eight ribs in the NCPAP group 12 h after extubation (*p* = 0.13).

**Table 2:** The primary outcomes between NCPAP treatment and NHFOV treatment.

**Variables**	**NCPAP**	**NHFOV**	***p*-value**
	**(*n* = 40)**	**(*n* = 40)**	
PRF, *n*	9	3	0.11
Reintubation within12 h after extubation, *n*	5	2	0.71
PaCO_2_, within 12 h after extubation, mmHg	43.6 ± 7.3	39.3 ± 5.8	0.05
OI, within 12 h after extubation, mmHg	207 ± 26	215 ± 29	0.19
Lung volumes on X-ray after extubation, median (IQR), rids	8 (7–8)	8 (8–8)	0.13

*NCPAP, nasal continuous positive airway pressure; NHFOV, nasal high-frequency oscillatory ventilation; PRF, postextubation respiratory failure; PaCO_2_, the partial pressures of arterial carbon dioxide; OI, oxygenation index*.

### Secondary Outcomes

There was no significant difference between the two groups in the total time under NIV or hospital stay (*p* > 0.05). The incidences of pneumothorax, mortality in the hospital, adverse hemodynamic effects, and treatment intolerance were similar between the two groups (*p* > 0.05). However, more infants with signs of discomfort and nasal trauma were observed in the NHFOV group, although the difference was not significant (Table 3).

**Table 3:** The secondary outcomes between NCPAP treatment and NHFOV treatment.

**Variables**	**NCPAP**	**NHFOV**	***p*-value**
	**(*n* = 40)**	**(*n* = 40)**	
Mean duration of NIV, median (IQR), days	2 (2–2)	2 (2–2.75)	0.58
Mean duration of hospital, median (IQR), days	9 (8–10)	8 (7.25–9)	0.50
Ventilation intolerance, *n*	2	3	1.00
The sign of discomfort, *n*	4	8	0.34
Pneumothorax, *n*	2	0	0.49
Nasal trauma, *n*	3	7	0.15
Adverse hemodynamic effect, *n*	0	1	1.00
Death in hospital, *n*	1	0	1.00

*NIV, noninvasive ventilation; NCPAP, nasal continuous positive airway pressure; NHFOV, nasal high-frequency oscillatory ventilation*.

## Discussion

To our knowledge, this was the first study to evaluate the effects of NHFOV vs. NCPAP in infants after CHS. Even though NHFOV has rarely been studied beyond neonatal age, it was shown to be feasible in infants *in vivo* without major complications (9). Infants who underwent CHS faced a greater risk of PRF than those who did not undergo heart surgery (10). NHFOV treatment was encouraged as the main postextubation respiratory support in preterm infants. Its efficacy and suitability have been reported in several preliminary studies (11, 12). We sought literature support on whether NHFOV therapy was also appropriate for our cardiac infantile patients (13). Since our patients were small infants, we hypothesized that NHFOV therapy may provide benefit to them. Therefore, we performed this study and found that NHFOV therapy was more efficient in improving CO_2_ cleaning, slightly improved pulmonary recruitment, and reduced the incidence of PRF and reintubation.

To our knowledge, NCPAP and invasive HFOV are two major respiratory support for infant respiratory failure (11). NHFOV has the combined advantages of these two respiratory support, including non-invasiveness, low tidal volume, and continuous pulmonary inflation. NHFOV seemed to be more effective in preventing PRF and reintubation than NCPAP. In a multicenter retrospective cohort study comparing NHFOV with NCPAP in preterm infants with respiratory distress syndrome (RDS), early use of NHFOV was found to be superior to NCPAP for reducing intubation (14). Chen et al. applied NHFOV as postextubation preventive respiratory support in preterm infants with RDS; it reduced reintubation and effectively removed CO_2_ within 6 h after extubation (3). Rescue NHFOV treatment was applied after NCPAP failure, which rapidly improved oxygenation and avoided intubation (15). In addition, NHFOV was useful both in early respiratory failure and in planned extubation with prolonged intubation (16). In preterm infants at high risk for respiratory failure, NHFOV was associated with significantly improving CO_2_ clearance and preventing respiratory failure (17). Our results were consistent with these previous studies. In our study, preventive NHFOV treatment was found to be more efficacious than NCPAP in preventing PRF and reintubation for infants who underwent CHS. However, there were inevitable problems during NHFOV treatment, and the risk of nasal trauma in NHFOV might be greater than that in NCPAP. The larger oscillations in NHFOV were superposed to mean airway pressure compared with NCPAP, which increased the pressure from nasal prongs to nasal skin. Therefore, the increasing incidence of nasal trauma might have reduced the comfort level under NHFOV therapy, which could result in adverse outcomes.

The exact mechanisms by which NHFOV is more efficacious than NCPAP in preventing PRF have not been fully explained. Our results showed that NHFOV would provide enough pulmonary recruitment and CO_2_ clearing compared with NCPAP, which might be a possible advantage to prevent PRF. Receiving higher pressure levels to maintain lung volume might benefit infants with NHFOV rather than NCPAP. This was because increasing the NCPAP level without any ventilation might increase the risk of gas trapping and pneumothorax (18). However, NHFOV had a low risk of gas trapping under high-pressure levels caused by oscillation ventilation and sufficient glottis expansion (19). Additionally, NHFOV does not induce inspiratory laryngeal narrowing or limit air into the gastrointestinal system, which helps to improve pulmonary ventilation (20). There were two reasons why NHFOV would effectively improve CO_2_ clearing. First, infants receiving NHFOV treatment faced a low risk of gas trapping-related CO_2_ accumulation. Second, the small oscillations in NCPAP could not reduce CO_2_ effectively (21). The larger oscillations in NHFOV were superposed to NCPAP, which helped CO_2_ diffusion in the upper respiratory tract (22, 23). Thus, NHFOV offered better pulmonary ventilation and CO_2_ clearing than NCPAP.

## Limitation

There were some limitations in this study. According to previous clinical experience, the NHFOV pressure could be set at values higher than those usually provided with NCPAP (3, 18). We designed different parameters for NCPAP and NHFOV treatment. Indeed, there was a limitation about the difference in pressure levels in the two groups, which may also have had an impact on the results. Although there were differences in our research results, there was no statistical significance, which may be due to the small sample size. We believe that the results of our study might reflect some clinical significance, so a large-sample, multicenter trial is urgently needed to confirm this conclusion. Last, our attending staff could not be totally blinded to the study group.

## Conclusions

This is the first study to assess NHFOV as postextubation respiratory support in infants after CHS. NHFOV therapy after extubation in infants after CHS was more efficient in improving CO_2_ cleaning than NCPAP therapy, but there was no difference in other outcomes (PRF, reintubation, oxygenation index, and pulmonary recruitment). NHFOV should be considered for use in infants with CHD after postoperative extubation.

## Data Availability Statement

The raw data supporting the conclusions of this article will be made available by the authors, without undue reservation.

## Ethics Statement

The studies involving human participants were reviewed and approved by the ethics committee of Fujian Maternity and Child Health Hospital (NO. 2020YJ181). Written informed consent to participate in this study was provided by the participants' legal guardian/next of kin.

## Author Contributions

H-LW and Y-RZ designed the study, performed the statistical analysis, participated in the operation, and drafted the manuscript. Y-QL, W-PX, and QC collected the clinical data. All authors read and approved the final manuscript.

## Conflict of Interest

The authors declare that the research was conducted in the absence of any commercial or financial relationships that could be construed as a potential conflict of interest.

## Publisher's Note

All claims expressed in this article are solely those of the authors and do not necessarily represent those of their affiliated organizations, or those of the publisher, the editors and the reviewers. Any product that may be evaluated in this article, or claim that may be made by its manufacturer, is not guaranteed or endorsed by the publisher.
